# Collectanea Medica, Consisting of Anecdotes, Facts, Extracts, Illustrations, Queries, Suggestions, &c.

**Published:** 1817-02

**Authors:** 


### COLLECTANEA MEDICA,

CONSISTING OF

### ANECDOTES, FACTS, EXTRACTS, ILLUSTRATIONS, QUERIES, SUGGESTIONS, &c.

RELATING TO THE

*History or the Art of Medicine, and the Auxiliary Sciences.*

Quicquid agunt medici, nostri farrago libelli.

### *Memoirs of the late Mr. Royston;* by Jos. Brookes, Esq.

WILLIAM ROYSTON, Esq. F.L.S. F.M.S. Bolt-court, and one of the Presidents of the Medical Society in Blenheim-street; died on Tuesday the 3d of December, 1816, aged 55, after being confined to his room for about three weeks.

This gentleman was formerly one of the editors of the London Medical and Physical Journal, and the projector of the Medical and Pharmaceutical Repository; of which last publication he continued till within a few months of his demise one of the enlightened editors.

Mr. R. possessed but a very indifferent state of health for the last four or five years, being frequently attacked with rheumatic

 and and nephritic complaints. About two years ago, Mr. R. was seized with a violent pain in his head; but this yielded to bleeding, cupping, &c. He was afterwards troubled with a pricking sensation in the ring and little fingers of the left hand, which continued to his death. He also laboured under a considerable difficulty in respiration, and was oppressed by flatus in the stomach to a most tormenting extent; and had, likewise, a great propensity to dozing for the last year or two of his life. In all these various maladies, Mr. R. was attended by his intimate friend, Dr. Bree, to whom Dr. Baillie, with his usual benevolence, joined his professional assistance. Dr. Curling, Messrs. Copeland, Dickenson (staff-surgeon), and Brookes, occasionally visited their afflicted friend, who was besides incessantly attended by Mr. Collins, surgeon in the India service.

Mr. R.'s principal complaint might be said to be Bulimia; but the consequences of indulgence were always great uneasiness in the stomach, flatulent distension, and long-continued eructations, accompanied by dyspnœa to a great degree. This vitiated state of the digestive organ was, by himself, attributed to the eating of ice in Bond-street, whilst warm from walking, about two years ago. To alleviate these distressing symptoms, Mr. R. was frequently induced to resort to the stimulus of brandy, mixed either with warm milk, or water, sugar, nutmeg, &c. which commonly afforded a temporary relief. By those prevailing affections, i. e. difficulty in respiration, flatulent distension, &c., Mr. R. was at length obliged to confine himself to the house; and, probably, to the same causes might be traced an extreme irritability of mind, remarked by his attendants, as also an occasional opposition on his part to the efforts of the physicians, who found themselves constrained to yield to the will of the patient in more instances than one. The internal means used were such as the variety and complication of the patient's sufferings seemed to call for, to which other auxiliaries, such as bleeding, leeches, a blister, frictions with Ung. Hydrargyri, &c. were superadded. All these, however, as will be hereafter seen, failed to produce the wished-for effect.

Mr. R., zealously attached to his friends, and by all those who knew him sincerely regarded, was a man of reading, of acknowledged professional talents, and of more than common acquirements in zoology, botany, painting, and music. He has left to be edited a work on Medical Electricity and Galvanism, which, according to the judgment of those who have seen it, is likely to do credit to its author.

This gentleman, the son of an opulent farmer, was born at Wigan, in Huntingdonshire: he studied in London in the year 1779, afterwards at Edinburgh in 1794; and began to practise at the place of his birth, which, however, not affording sufficient scope for exertion, he came, by the advice of Dr. Glynn and other medical friends, to the metropolis in the year 1806, and established himself in Prince's-street, Cavendish-square, where he died.

About

About a year ago, Mr. R. turned his attention more particularly to electricity and galvanism, and had a very elegant and appropriate apparatus, constructed by Cuthbertson, with which he experienced the satisfaction of removing many very serious maladies, such as amaurosis, cataract, contraction of the arm, asthma, &c.

### *Dissection.*

On Wednesday, the 4th of December, Mr. Brookes examined the body in the presence of Dr. Baillie, (Dr. Bree being engaged,) and of Dr. Curling, Messrs. Dickenson, Copeland, Collins, Morgan, and Roscoe, (the two last pupils of Mr. Brookes's,) and Mr. Toke, apprentice to Mr. R.

On opening the abdomen, the liver presented itself much diseased, hard, rather less in size than naturally, of a lighter red colour, tuberculated; in its interior part nearly as firm as in its exterior. The gall-bladder and biliary ducts were not at all diseased.

The stomach was small, nearly empty, except at the cardiac extremity, contracted across the middle; and, on being divided on its anterior surface lengthwise, appeared of a dark reddish hue, with the pylorus morbidly thickened.

The spleen was much firmer than usual, its parenchymatous structure retaining the same compact texture throughout. The artery was much diseased, its coats very opake and verging towards ossification.

The pancreas partook of a similarly indurated structure.

The other abdominal viscera were not at all altered, but the bladder was thickened in its coats, and somewhat inflamed at the termination of the ureters.

The prostate gland was like the other diseased viscera, firmer in its structure than when in a healthy state, rather larger, and its middle lobe projecting into the bladder.

The kidneys were strikingly diseased, being not more than half of their usual size, exhibiting the morbid features of the other viscera particularized, the cortical part by no means of that ruddy colour which is their usual appearance; the tubulary portion and the tubuli uriniferi more than commonly distinct and obvious.

*The Thorax.*—On removing the sternum and cartilages of the ribs, the left lung was found but little altered, nevertheless inflammation had taken place and produced some trifling adhesions between the pleura pulmonalis, and costalis. The right lung, on the contrary, was very much and recently inflamed, suffused over its surface with coagulable lymph, and floating as it were in about a pint of serum. The division of the pericardium offered to our view a large heart, having on the middle of the pulmonic ventricle an opake spot about the size of a shilling, denoting carditis, that had existed at some remote period. There was another spot of the same kind at the very apex of the aortic ventricle. The apex possessed a rotundity not natural. All the valves of this viscus were firmer than usual, but not at all ossified.

The

*The Head.*—On examining the brain, this organ offered but little to interest the physician, further than that the plexus choroides in the left lateral ventricle was found to have a few hydatides attached to it, but of no great size; and, as is generally observable in a subject of nearly sixty years of age, the cerebral and vertebral arteries, with the vasilary, changed in structure, opake, having lost their contractility, and fast approaching to ossification, and in this resembling the condition of the splenic artery before noticed. There was half an ounce of fluid in each lateral ventricle. In other respects, nothing occurred worthy of description.

### *Reflections.*

From whence then did this accumulation of disease arise in an individual of temperate habits, with whom I have frequently associated in parties where the prevailing conviviality might have tempted the most abstemious man to depart somewhat from his ordinary rules? Even on such occasions, my late friend never appeared in the least disposed to exceed: in other respects, perfectly regular, rising early in the morning, and, during the day, walking ten or more miles. Could mental anxiety have contributed to produce such morbid changes throughout the constitution? Is it possible, that disappointment in those views with which Mr. R. had been induced to settle in the capital could have occasioned such a train of evils?—I must own, that to all these questions, I would not hesitate to answer in the affirmative.

---

*Case of Puerperal Fever, in which the Uterus and Spleen were principally affected.* By Hugh Ley, M.D. Physician-Accoucheur to the Westminster Lying-in Hospital.—(From the Medical Transactions.)

Ann Taylor, aged about 28 years, of a dark swarthy complexion, and exhibiting signs of the melancholic temperament, was admitted into the Westminster Lying-in Hospital, on the evening of the 25th of September, 1814. Little or no progress had been made in her labour; she was delivered, however, in three hours after her admission, by Mr. King, an industrious and intelligent student. Nothing untoward occurred in her delivery. Her labour was, in every respect, perfectly natural; the dilatation of the os uteri being rapidly progressive, the presentation most favourable, and the external parts easily "dilatable." As soon as the division of the funis had been effected, and the infant delivered to the ordinary attendant, it was ascertained that the placenta had been separated by the contractions of the uterus, and was already lodged in the vagina. In a few minutes it was wholly expelled by the natural efforts of the woman, no assistance having been given by the funis. An opiate having been administered, she was put to bed. The ordinary treatment of the house was had recourse to. She was confined to a low diet, consisting solely of vegetable decoctions, coctions, and strict abstinence from the use of fermented liquors was enjoined. On the following morning, as she complained of a slightly painful affection of the stomach, her bowels were opened by a solution of the sulphate of magnesia, in the infusion of senna; and by this she was completely relieved.

On the morning of the 28th, my attendance was desired at the hospital on account of the serious indisposition of this woman. She complained of intense pain over the whole abdomen, and especially in the umbilical region. It was acute and permanent, and upon making even inconsiderable pressure over any part of the abdominal cavity, the pain became excruciating and insupportable. There was, at the same time, some slight fulness of the abdomen, without, however, any considerable degree of tension.

These symptoms of local disease were accompanied with much constitutional affection. She had been attacked on the day preceding with cold chills, amounting even to shivering, succeeded by, and alternating with, irregular flushes of heat, head-ache, pain in the loins, and wandering pains of the limbs. Her head-ach now continued; she complained of restlessness, with great prostration of strength and depression of spirits, her countenance being strongly impressed with anxiety. The pulse was frequent, exceeding 120 in a minute, of contracted diameter, and hard: the respiration hurried and anxious. Her thirst was insatiable; the tongue, towards its middle, had a thick white tenacious crust, the edges being of a bright red colour. She had some degree of nausea, without, however, any effort to vomit; and her bowels, which had been previously confined, were now under the influence of a cathartic, which had produced several liquid evacuations. These were, however, in small quantity, dark-coloured, and highly offensive. The skin was dry and pungently hot. The urine was discharged without difficulty, but in small quantity; it was high-coloured, and deposited a copious sediment. Her milk, of which she had little on the preceding day, now wholly disappeared; and the lochial discharge was inconsiderable, and slightly offensive.

These symptoms appearing to justify and require the use of the lancet, I directed her to be bled immediately. I remained with her until this had been accomplished, keeping my finger upon the artery of the opposite wrist during the operation. The frequency of the pulse was scarcely altered as the evacuation proceeded, though its first effect was evidently to increase its volume. Having taken from 12 to 14 ounces of blood, as I found the pulse becoming languid, without, however, any other signs of syncope, I directed the arm to be tied up: as I conceived the evacuations from her bowels, although frequent, to be insufficient, and affording evidence of the undue retention of feculent discharges, I gave instructions for the immediate exhibition of a strong cathartic, the basis of which was calomel; and this to be followed up by divided doses of the sulphate of magnesia; and unless, in the evening, the pain should have completely ceased, I desired that a blister might be applied.

On

On the following morning her symptoms were much relieved; she scarcely at all complained of internal pain, and she could shift her position without inconvenience. The frequency of pulse was diminished, as were also the thirst and heat of surface; the tongue was more clean, and her bowels had been well opened,—highly discoloured and offensive evacuations having been produced by the medicines administered; the alvine discharges, too, had now become feculent and somewhat scybalous. She had still, however, no milk, and the lochia were still in trifling quantity. She had, moreover, passed a night of restlessness, which she ascribed to the irritation of the blister.

By repeating small doses of laxative medicines, especially the salines, she so far recovered, that I felt justified in taking my leave, giving general directions to pay strict attention to her bowels, and in case of a relapse, to apply to Dr. Gooch, whose month of attendance had commenced.

Having, on the morning of the 4th of October, occasion to visit the hospital on account of the engagements of Dr. Gooch, I was requested by him to see two cases, to which he had been called on the preceding evening. One of these I found to be my former patient, whom I had supposed to be by this time fully recovered. Thinking her case desperate, I hastened to request the advice of our consulting physician, Dr. Maton, who readily consented to meet me in the evening. We found her labouring under much constitutional irritation, without any *apparent* proportional local affection. She complained of pain in no part, and could bear considerable pressure on every part of the abdomen, which was little, if at all, enlarged: she had some heat of skin, with moisture which approached to clamminess. Her pulse was rapid, small, and compressible; her respiration frequent, anxious, and even somewhat laborious; her countenance and eyes were suffused with a yellow colour, her cheeks exhibiting the circumscribed redness of hectic; her tongue was foul, and, although she did not complain of thirst, she took fluids in small quantity when offered to her, and seemed to be refreshed by them. Her stomach had become extremely irritable, rejecting every thing which she had taken in the way of nourishment, to which, indeed, she had little inclination.

Upon making inquiries as to her state in the interval between my attendance and the evening when Dr. Gooch was sent for, I was informed, that, though she had kept her bed, she had made no complaints, had not refused to take food, had been cheerful, and even jocular; but that her bowels had been obstinately constipated.

For the symptoms under which she now laboured we had difficulty in accounting: they seemed to indicate the existence of some visceral affection, but to what organ to refer as the seat of the disease, in the absence of all pathognomonic symptoms to direct our judgment, we were unable to determine. We had reason to suspect, however, the existence of some biliary obstruction. She had, indeed, complained of no pain in the region of the liver, but her her countenance exhibited a jaundiced appearance; her urine had a bilious tinge, and her bowels had resisted the most powerful purgatives; even 10 grains of calomel, which had been administered on the preceding evening, had produced little or no effect. It was now, therefore, judged expedient to keep up an action upon her bowels, if possible; and with this view, as her stomach was so irritable that neither nourishment nor medicines of any bulk could be retained, and, as it was found, in this case, that calomel, failing to produce any purgative effect, had increased the general irritation of the system, pills, the basis of which was the compound extract of colocynth, were directed to be given till they produced their effect.

On the following morning, to our surprise, we found her sinking: the pulse had become incalculably rapid, indistinct, and occasionally intermitting; the surface was moist and clammy, the extremities cold; the respiration had become laborious, the eyes sunk in their sockets, the intellect wavering, the lips livid, and the countenance ghastly. Cordials were administered in vain—she expired on the same evening.

*Dissection.*—On opening the general cavity of the abdomen, few marks of inflammation could be immediately detected; and, indeed, had not the attention been particularly directed, by the preceding symptoms, to its investing membrane, the very slight turgescence of vessels which this exhibited would altogether have eluded observation.

The serum contained in the cavity of the peritonæum did not exceed an ounce, and this not assuming the appearance so commonly observed in the puerperal peritonitis, but resembling that which is effused under other circumstances, or into those other cavities of the body, which are lined by a serous membrane, possessing, indeed, a community of general properties with that serum, which may be obtained by spontaneous separation from a portion of the general circulating mass.

On tracing the course of the intestines, no preternatural thickening of their tunics, no adhesion between their contiguous parts, appeared; no coagulable lymph had been effused upon their surfaces, nor were there any shreds of the same substance floating in the serum. In one part of the intestinal tube, however, there was a slight exception to its generally healthy aspect: the sigmoid flexure of the colon exhibited unequivocal marks of its having been the subject of slight inflammation; it adhered slightly to a part, which, although much altered in its appearance and structure, was recognised as the left Fallopian tube, and was slightly red, especially at that part where the two layers of peritonæum divaricate to cover that portion of the canal. The agglutination, however, of these contiguous parts could be with facility separated by the finger: it was evidently of recent formation, and might, perhaps, be considered rather as an extension by contiguous sympathy (to adopt the phraseology of Mr. John Hunter) of a disease originally affecting the Fallopian tube of that side, than as a primary disease of of the part itself. It was in the uterus, and its appendages on the left side, that we found the principal seat of the disease, which, to appearance, proved destructive to the patient.

The uterus, which was scarcely more enlarged than we might expect to find it at such a period after delivery, exhibited externally slight marks of inflammation; its peritonæal covering was more red, and somewhat thicker than usual, especially towards that part of the fundus where the left Fallopian tube emerges from the uterus. The thickness of its parietes was not much augmented, the muscular structure was more pallid than is common, whilst the principal vessels, which could be observed ramifying through its substance, were enlarged and turgid. On laying open the cavity of the uterus, a striking change of organic structure exhibited itself: no appearance of mucous membrane remained; the whole surface had assumed a gangrenous appearance, was extremely irregular, and of a dark livid or greenish hue; and these appearances were accompanied with considerable fœtor. Towards the fundus, which appeared to have suffered most, were found portions of coagulated blood, adhering firmly to the uterus, and much resembling polypi. One of the largest of these substances occupied the left angle of the cavity of the uterus, covering, as nearly as we could judge in such an altered state of parts, the exit of the Fallopian tube on that side: this, with the ovary on the same side, were materially altered in their structure; they had lost their natural character, and appeared now to consist only of a cellular tissue, whose cavities had been filled by a serous exudation. In separating the uterus from its connexions, with a view to a more deliberate and accurate examination of its state, the incision was unfortunately not made sufficiently low in the pelvis to include the os uteri. Of its state, therefore, we had no means of obtaining accurate information. It is reasonable to conclude, however, that it had partaken of the disease, since the whole of the cavity of the uterus was affected, and even in that part of the vagina which could be submitted to ocular inspection, by the separation from each other of the labia, some degree of livid discoloration could be observed. It is possible, however, that this might have arisen from the passage over its surface of a fluid formed in the uterus, and not altogether from the effect of disease attacking at least the inferior part of the vagina.

The liver was of its natural size, and healthy in its structure. The gall-bladder was not at all distended with bile, although it contained a small quantity; and there were no biliary concretions found either in it or in the cystic, hepatic, or common bile-duct.

The pancreas and stomach appeared to have undergone no morbid change.

The spleen, throughout the whole extent of its convex surface, adhered to that portion of peritonæum which, situated opposite to it, lines the left hypochondrium. In attempting to destroy this connexion by the hand, it was stripped of that portion of its peritonæal covering. Internally this viscus had undergone a very extraordinary traordinary morbid alteration: its texture was so changed as to resemble an extremely soft piece of sponge, of which the cells had been filled by an intimate mixture of pus and of grumous blood. On placing it in water, for the purpose of washing out this matter, the whole organ exhibited an appearance somewhat resembling that of a lung exposed to long-continued maceration, to illustrate its great vascularity. Innumerable ramifications of vessels, of which the connecting medium had been destroyed, floated separately in the water, rising from every point of the superficies of the organ. These vessels, in their extreme minuteness, and the immensity of their number, might be compared, with justice, to the small fibres which compose the down of the swan; nor did this exceed in softness the impression which was occasioned by passing the hand over the surface of the spleen, which now appeared to consist wholly of a congeries of minute vessels, deprived, by interstitial or ulcerative absorption, of their connecting cellular membrane, and of the parenchymatous substance, if such exist, independently of the vascular and nervous systems, and the connecting membrane. The contents of this spongy mass having been removed by repeated washing, something like an attempt at the formation of cavities to contain the matter manifested itself; no regular cyst, however, had been formed. The parietes of the abscesses (if abscesses they could be called), were imperfect and pervious, being composed of blood-vessels running in every conceivable direction, and forming a thick and beautiful plexus or net-work; each vessel, however, remaining as unconnected and distinct as if all its surrounding connexions had been removed by the most careful and accurate dissection. I know of no structure in the human body to which I can refer, to illustrate, by its resemblance, the appearance which these cavities assumed, unless it be the internal formation of the heart. The fleshy columns of this organ "compose a beautiful, intricate, and irregular net-work;" and certainly bear a distant resemblance to the arrangement of vessels which I have described, although the latter were infinitely more numerous and minute. The ulcerative process seemed, in some instances, to have extended itself even to the coats of the vessels, some of the largest of which appeared to be eroded.

The urinary organs had not been at all subjected to the influence of this disease. The kidneys, however, afforded us an opportunity of observing a curious instance of the "anatomia lusoria." On the first examination of them *in situ*, they appeared to have nothing peculiar in their form or structure. When, however, the surrounding parts had been removed, it was discovered, upon more accurate inspection, that the kidneys, united on the spine by their inferior extremities, formed one continuous body of a semilunar form; and this, from its general resemblance in shape to the shoe of the horse, has, by some eminent anatomists who have in their possession instances of a similar structure, been denominated the 'horse-shoe kidney.' The artery on each side entered at the superior parts or cornua of the kidney, while the vein and ureter passed passed out very near to each other, and at a part which would answer to the proper situation in a kidney of the ordinary form.

The bladder was of its natural size, not in the least distended, and of a perfectly healthy aspect.

It did not appear that the thoracic viscera had been affected by *this* disease. The quantity of serum contained in the pericardium scarcely exceeded what is usually found. The heart appeared healthy in size and texture; the lungs had undergone no morbid change; the pleura pulmonalis of the left side adhered by every part of its surface to the contiguous part of the sac, which lines the ribs and intercostal muscles. These unnatural adhesions, however, appeared to be the effect of former disease.

The brain was not examined.

*Observations.*—Having now completed the general outline of a case, which has appeared to those in whose judgment I have confidence to be not altogether devoid of interest, it may not be foreign from the leading object of my communication, to direct the attention to those peculiarities by which it is characterized, although the necessarily circumscribed limits of a paper of this kind will prevent my entering so minutely into details as either I could wish, or may appear necessary, for the complete elucidation of those points in pathology which it is calculated to illustrate.

The case seems to afford proof and illustration of a fact, which reasoning and observation conspire to prove, that puerperal inflammatory diseases are by no means essentially connected with the tediousness and difficulty of labour; and, indeed, were I entitled to draw any general conclusion from my own limited experience, I should not hesitate to assert, that, in those who, from causes, external or internal, natural or artificial, local or general, have gone through, with unusual celerity, the various stages of parturition, such affections are more frequent in their occurrence, more violent in their operation, and more fatal in their event, than in those whose sufferings have been long protracted and severe in the course of delivery. But upon this point, which might lead me into speculative inquiries of some length, obvious circumstances will oblige me to be brief. I shall venture to consider the apophthegm. common in the lying-in room, that " women have a better getting up after slow than after quick labours," as founded on accurate observations, and by no means as a prejudice of the " profanum vulgus," unsupported by evidence, and consequently unworthy of attention;—and it will be removing one great obstacle to the admission of the truth of this opinion, if it can be shown that the assumption (if that can be termed an assumption, which is a general though vulgar induction from experience) can do *no* harm, but *must* have a beneficial influence on the practice of those engaged in obstetrical pursuits. This it would not be difficult to prove to the satisfaction of those whose minds, uninfluenced by the bias of preconceived opinions, are open to the impressions of truth. To such, a belief in the existence of a natural relation between the celerity of labour and the occurrence of puerperal affections of a febrile and inflammatory kind, cannot but inculcate caution in the management of parturient and puerperal women: it will prevent an over anxiety in the progress of labour for its speedy termination, and put an end to the various modifications of improper practice founded on such solicitude; nor can it fail to urge them to an attentive and cautious observance of the consequences of cases of unusually quick labours. In these, which have been hitherto too much neglected, the baneful influence of an unrestricted regimen is more to be dreaded than even in those in the progress of which the woman has suffered long and violently. Such cases too, as they arise from causes, either external or internal, artificial or natural, become either the direct causes or the signs of future disease; the extent and kind of morbid affection being also, in some measure, influenced by the cause, in proportion as this may be local or general. These very general statements, however, will become more obvious and intelligible, in their application to the individual case in which these reflections have originated; and, in doing this, it will be necessary to bear in mind both the general habit of the patient, and the circumstances of her labour.

The appearance of this woman, it will be recollected, indicated that languor of habit which is characteristic of the melancholic temperament.—The indisposition to febrile affections which usually accompanies this habit of body, though it did not wholly prevent the operation of a common cause in the production of puerperal fever, modified the attending symptoms; for, notwithstanding that the inflammation of the investing membrane of the abdomen was extensive and violent, the concomitant fever was not so severe as I have frequently witnessed, and appeared to be checked *in limine* by the means adopted for its cure.

From the general aspect of the patient, then, there appeared no reason for extraordinary caution in guarding against an attack of puerperal fever. But in the extreme celerity of the labour, and especially from the quickness with which the uterus, impatient to be freed from its contents, had, by its contractions, separated the placenta, there appeared reason (supposing the connexion which I have endeavoured to trace to have been previously ascertained) to infer the existence of such an *irritable* state of the uterine system, as would be sufficient, if not to constitute a strong predisposition to puerperal fever, at least so to modify the disease, that the violence of its attack would be directed particularly upon the uterus and its appendages: and such appears to have been the case in the instance before us.

It is an extraordinary feature of the present case, that two organs so remote from each other, and possessing, apparently, so little mutual affinity either in structure or function, should have been so particularly affected, whilst all the intervening organs had escaped the destructive influence of a disease, which had evidently been common to the whole. The co-existence of morbid affections of these two organs, however, is by no means of unfrequent occurrence; currence; and, indeed, it is a curious fact, that few cases of diseased spleen in the female have been related by Morgagni, in which there was not some concomitant affection of the uterus or its appendages. Such cases ought, perhaps, to be considered only as coincidences, and wholly insufficient to warrant the conclusion, that there is any natural alliance between these two organs; yet still the fact is, to a certain extent, interesting, and deserving of attention.

It may be proper here to notice a difference of opinion which has subsisted, with regard to the actual state of the uterus in this case. Some, more conversant with morbid dissections than myself, had no doubt that gangrene had occurred. I am not convinced, however, that this was absolutely the case; and, in supporting the opposite opinion, whilst I am sanctioned by the authority of an eminent teacher of midwifery, to whom I showed the part soon after its removal from the body, I am influenced by the following reasons: first, because, considering the extreme unfrequency of gangrene (which I conceive to involve a total abolition of vital power) of the uterus, I am unwilling, without strong and irrefragable proof, to admit its existence; secondly, because, in the case before us, there was not that diminution of the solidity of texture which characterises gangrene; and, thirdly, because I think the phenomena explicable upon another supposition; this being founded on the effusion and subsequent putrefaction of lymph, and of blood in its aggregate state, effused upon the internal surface of the uterus. Such a supposition seems to account satisfactorily for the irregularity of surface, with the gangrenous fœtor and lividity which were observed. But, whatever opinion may be entertained upon this point, the appearances which, in the account of the dissection, I have endeavoured faithfully to describe, without involving any discussion of their cause, were abundantly sufficient to justify the conclusion, that the uterus and its appendages had suffered from a very high degree of inflammation, the combined effect of which, with that of the affection of the spleen, without producing any decided characteristic signs of the organic affections, gradually undermined the powers of the constitution, and ended only with the destruction of the patient.

Of the state of the spleen, as illustrating some of the general positions and principles established by Dr. Carmichael Smith, in his valuable paper on Inflammation, I must refrain from treating at any length, howsoever interesting it might be to examine those peculiarities of inflammation of the spleen, which may be ascribed to two great sources of diversity in the phenomena of inflammation pointed out by Dr. S.—"function" and "natural structure." I must here, therefore, content myself, without attempting to explain their connexion, with referring to the former source of difference the symptoms of hepatic affection, and to the latter the freedom from pain, and the peculiarity of termination, (which last I believe to be rather general than accidental) exhibited on the dissection of this woman.

Even upon the connexion of inflammation of the substance of the spleen with puerperal fever, I shall be unable to dwell long. This relation, which seems to have escaped, in great measure, the particular attention of pathologists, has not altogether eluded observation. That inflammation of the peritonæal covering of this organ may constitute a part of the peritonitis puerperalis, is a fact well ascertained, and not unfrequently observed. The alteration, however, of its internal structure had been rarely noticed, until Gastellier, during the short time that he had the care of the Hospice de la Maternité, had an opportunity of observing it. Having given the general result of his observations on the dissection of those who died of the epidemic which he has described, he says, "La rate en a été *souvent* frappée, mais une fois entr'autres elle a été entièrement détruite, entièrement fondue; il n'en restoit aucune trace, si non un foyer de fluide sanieux, dans la région, et en place de cet organe."

In another case of puerperal fever which I had recently an opportunity of examining, the spleen appeared to have undergone a morbid alteration, similar in kind, but differing much in degree, from that observed in the instance which forms the subject of this communication. The peritonæal covering could, with the greatest facility, be stripped from the whole of its convex surface; and the interstitial substance had been somewhat, though not extensively, removed. This, however, had been effected to such an extent, that the spleen, possessing but little of its usual solidity, had assumed the texture of sponge very unusually soft, and was beginning to exhibit that flocculent appearance which, in a former part of my paper, I have endeavoured to describe.

Whether this morbid affection would have gone on to the degree of disorganization already described, it is, perhaps, not easy to determine; but, since the appearances much resembled those parts of the spleen which were the least affected, in the case which I have detailed, it seems probable that such would have been the event, had the patient's life been protracted sufficiently long; and, if so, we must consider it as the beginning of a disease, of which this and Gastellier's case (and it was with the view of comparing these cases that I quoted the words of Gastellier) form the extremes of commencement and termination, while the appearances on the dissection of Taylor point out that intermediate stage, or extent of the disease, in which the removal of the vascular structure had yet only commenced.

My own experience does not enable me to state, with any precision, what is the relative frequency of affections of the spleen in puerperal fever; nor would such knowledge be of much practical utility, since it would scarcely influence the practice. In the cases which have fallen under my observation, I have had reason to think that they constitute no unfrequent occurrences. We know, however, that it is a characteristic feature of some endemic diseases, to be accompanied, in the majority of instances, by an affection of some particular organ; and it is not impossible but that that to this very general principle should be referred the frequent occurrence of affection of the spleen, in those cases of puerperal fever which I have lately seen, as well as in those which occurred during the two months of M. Gastellier's attendance at the Hospice de la Maternité.

It now only remains that I advert very briefly to the unnatural formation of the kidney, which, however, as it was obviously congenital, scarcely admits of discussion. It does not seem to throw any light upon the natural structure, function, or diseases of this organ, and is, therefore, no more deserving of attention than as recording one of those instances of deviation from the ordinary course of the operations of nature, with which we occasionally meet. We are entitled to infer, that it was an original malformation, both from its internal structure and the disposition of its vessels. The arteries had not their usual relative situation with respect to the other vessels, and at the part where it might have been supposed that an unnatural union had been effected by former disease, the peritonæum was dissected back with care, to ascertain if any process of this membrane dipped down between what might have been considered as the natural division of two organs; but nothing of this kind could be detected. The whole consisted of a continuity of substance identical in its appearance and properties, and having no intervention of membrane, or variation of structure, to mark the situation where we might have expected to find traces of former separation.

